# Unexpected Gaucher disease in a case of steroid-resistant nephrotic syndrome

**DOI:** 10.1007/s00467-025-06991-z

**Published:** 2025-10-08

**Authors:** Mona Hamed Gehad, Doaa Mohammed Youssef, Wesam A. Mokhtar, Manar M. Fathy, Amal Gohary

**Affiliations:** Department of Pediatrics, Faculty of Medicine, Zagazig University, Al Sharqia Governorate, Zagazig City, Egypt

**Keywords:** Gaucher disease, Nephrotic syndrome, FSGS, Enzyme replacement therapy

## Abstract

Gaucher disease (GD), the most common lysosomal storage disorder worldwide, should be considered in children presenting with unexplained hepatosplenomegaly and cytopenia. Kidney involvement is rare, and nephrotic syndrome constitutes an uncommon complication. We describe a 15-month-old female, the first child of consanguineous parents, who initially presented with infantile nephrotic syndrome. Kidney biopsy revealed focal segmental glomerulosclerosis (FSGS). The patient had the first corticosteroid treatment without any improvement. Mycophenolate mofetil was then added, resulting in partial remission. The patient subsequently developed pancytopenia, progressive stridor, hepatosplenomegaly, and recurrent seizures. Molecular genetic testing confirmed GD. Enzyme replacement therapy (ERT) improved cytopenia, seizure control, and organomegaly, as well as uremic manifestations; however, progression to kidney failure was not reversed.

## Case presentation

A previously healthy 15-month-old female, the first child of consanguineous parents from a moderate socioeconomic background, was admitted to the pediatric nephrology unit with generalized body swelling. Clinical examination revealed generalized edema and moderate ascites. Her prenatal, past medical, and family histories were unremarkable. Developmentally, she demonstrated delayed motor milestones but maintained normal cognitive function.

Initial laboratory investigations showed hypoalbuminemia (1.4 g/dL), nephrotic-range proteinuria, and preserved kidney function (serum creatinine 0.18 mg/dL; blood urea nitrogen [BUN] 13 mg/dL). Liver enzymes were within normal limits (alanine aminotransferase [ALT] 8 IU/L, aspartate aminotransferase [AST] 18 IU/L). TORCH screening was negative and complement C3 level was within the reference range (1.2 g/L; reference: 0.9–1.8 g/L). Kidney ultrasound demonstrated normal-sized kidneys with increased cortical echogenicity and preserved corticomedullary differentiation, consistent with Grade 2 nephropathy.

The patient was diagnosed with infantile nephrotic syndrome and commenced on corticosteroid therapy (prednisolone 60 mg/m^2^/day). After six weeks without clinical or biochemical improvement, she was classified as having steroid-resistant nephrotic syndrome (SRNS). Kidney biopsy demonstrated focal segmental glomerulosclerosis (FSGS), not otherwise specified (classic variant), with global glomerulosclerosis involving 15% of glomeruli and negative immunohistochemical staining.

Cyclosporine A (3 mg/kg/day) was initiated as second-line therapy; however, treatment was discontinued due to the development of hypertension and worsening kidney function. The regimen was subsequently switched to mycophenolate mofetil (600 mg/m^2^), which resulted in improvement of edema and a partial reduction in proteinuria.

Despite partial remission, the patient required prolonged hospitalization due to recurrent infections, including multiple episodes of gastroenteritis and lower respiratory tract infections. Over the following weeks, she developed progressive kidney impairment characterized by oliguria, abdominal distension, and fluid overload. Echocardiography revealed mild pericardial effusion. A diethylenetriaminepentaacetic acid (DTPA) scan confirmed Stage 5 chronic kidney disease, necessitating initiation of regular hemodialysis three times per week.

Six months after the initial admission, the patient was transferred to the intensive care unit with acute gastroenteritis, recurrent seizures, and a sepsis-like presentation. On examination, she appeared critically ill with Grade 2 stridor and hepatosplenomegaly. Laboratory findings demonstrated pancytopenia (hemoglobin 7.8 g/dL, leukocytes 4,000/μL, platelets 25,000/μL). Despite ongoing hemodialysis, kidney function had deteriorated (serum creatinine 3.2 mg/dL, BUN 57 mg/dL). Hypoalbuminemia persisted (1.4 g/dL). Although liver enzymes remained within normal limits, coagulation studies indicated coagulopathy (INR 1.3, prothrombin time [PT] 13.5 s, partial thromboplastin time [PTT] 60 s). The erythrocyte sedimentation rate was elevated (60 mm in the first hour), while C-reactive protein was negative. Bone marrow biopsy was inconclusive, revealing normocellular marrow without evidence of abnormal infiltration. Central nervous system infection was excluded by a comprehensive negative virology and immunology panel.

**Fig. 1:**
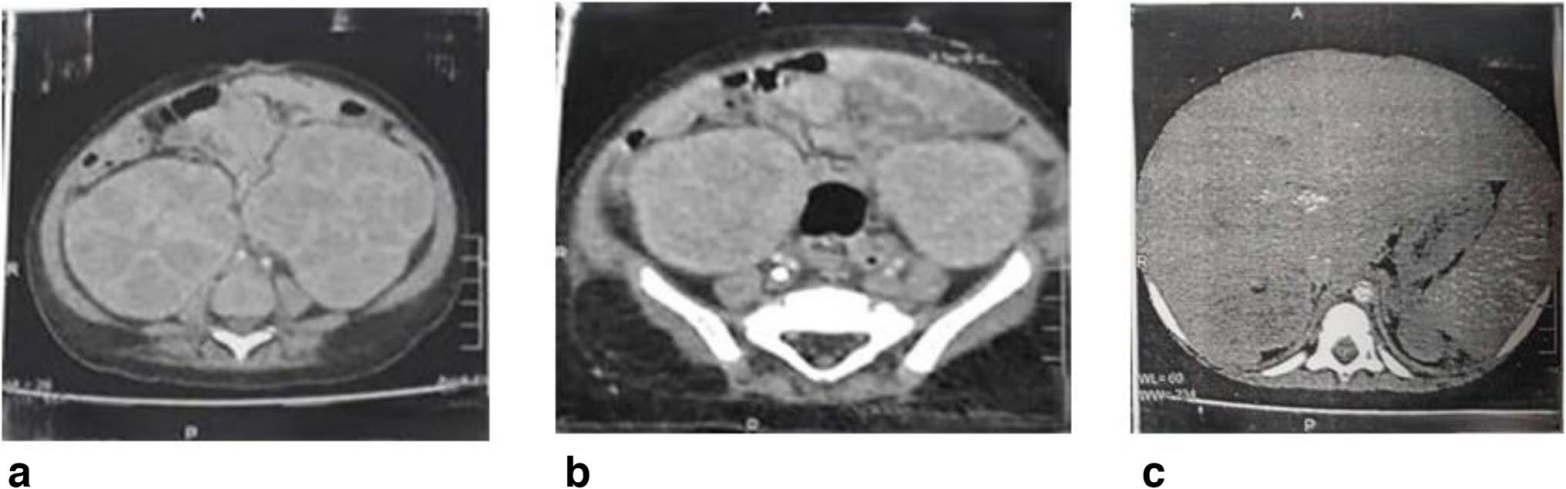
**a** CT images of abdomen and pelvis with IV contrast showing swollen renal parenchyma with cortical enhancement. **b** Bilateral kidney enlargement that extends down to sacroiliac joint with preserved kidney outlines. **c** CT scan showing diffusely enlarged liver and spleen. CT, computed tomography

Computed tomography of the neck, chest, and abdomen revealed bilaterally enlarged kidneys (right: 10.8 × 8 cm; left: 19 × 8.5 cm) and hepatosplenomegaly (liver 12 cm; spleen 8 cm) (Fig. 1). Both the brain MRI and the EEG were unremarkable.

Given the constellation of pancytopenia, coagulopathy, organomegaly, progressive kidney dysfunction, and neurological symptoms, a metabolic storage disorder was suspected. Genetic analysis identified a homozygous missense variant of uncertain significance (Class 3) in the *GBA* gene: c.1222A > T (p.Thr408Ser). Biomarker analysis revealed markedly elevated lyso-Gb1 levels (13.9 ng/mL; reference ≤ 6.8 ng/mL), and beta-glucocerebrosidase enzyme activity was severely reduced (< 0.29 μmol/L/h; reference ≥ 4.1 μmol/L/h), confirming the diagnosis of autosomal recessive Gaucher disease (GD).

The c.1222A > T (p. Thr408Ser) substitution results in the replacement of threonine by serine at codon 408 in exon 8 of the *GBA* gene. To the best of our knowledge, this represents a novel pathogenic variant not previously reported in the literature.

Enzyme replacement therapy (ERT) was initiated at a dose of 60 units/kg every other week. This resulted in improvements in cytopenia, seizure control, hepatosplenomegaly, and uremic manifestations; however, the patient remained dependent on regular hemodialysis.

## Discussion

We describe a rare case of GD in an infant who initially presented with SRNS and progressive chronic kidney disease—an unusual kidney manifestation of a metabolic lipid storage disorder. The subsequent diagnosis of GD was established by genetic and enzymatic testing, and ERT resulted in significant clinical improvement.

GD is the most common lysosomal storage disorder, caused by a deficiency of the enzyme glucocerebrosidase and inherited in an autosomal recessive pattern. It is traditionally classified into three subtypes [1]. Our patient demonstrated clinical features consistent with a neuronopathic form, including pancytopenia, hepatosplenomegaly, and central nervous system involvement such as motor delay, Grade 2 stridor, and recurrent seizures requiring antiepileptic therapy.

The neurological profile of Type 2 GD is characterized by early-onset, rapidly progressive brainstem dysfunction, with manifestations including epilepsy, myoclonus, trismus, stridor, and progressive microcephaly [2]. The combination of systemic and neurological manifestations in our patient raised suspicion for either Type 2 or Type 3 GD.

Kidney involvement in GD is exceptionally rare. When present, histopathological findings typically reveal Gaucher cells in glomeruli or the interstitium, usually as incidental findings. Reported kidney manifestations include hematuria, proteinuria, and glomerulopathies such as membranoproliferative glomerulonephritis (MPGN) and FSGS [3, 4]. Unlike previous cases, where glomerulopathy developed after an established GD diagnosis, our patient initially presented with SRNS/FSGS before recognition of GD. This raises the possibility that lysosomal accumulation of glucosylceramide or glucosylsphingosine may induce podocyte injury, activate pro-fibrotic pathways, and promote glomerulosclerosis.

The association between GD and glomerular pathology has been highlighted in earlier reports. One case described a 35-year-old male with GD who presented with hepatosplenomegaly, ascites, bone destruction, myelofibrosis, and MPGN, and who showed clinical improvement with oral prednisone and mycophenolate mofetil [4]. Similarly, our patient demonstrated partial remission with mycophenolate. In another case reported by Al-Bderat et al. [3], a 5-year-old male with developmental delay developed SRNS, with kidney biopsy confirming FSGS and eventual progression to kidney failure requiring dialysis. The later onset of cytopenia and organomegaly confirmed GD with a bone marrow biopsy showing lipid-laden histiocytes and reduced glucocerebrosidase activity [3]. These findings parallel our patient’s clinical course. Of note, neither kidney biopsy nor bone marrow in our case revealed Gaucher cells, likely reflecting their sparse distribution in tissue samples and highlighting the limitations of biopsy. Currently, diagnosis can be reliably achieved through enzymatic assays in leukocytes, without requiring tissue biopsy [5]. Thus, the coexistence of nephrotic syndrome, organomegaly, and hematologic abnormalities should prompt consideration of an underlying metabolic or genetic disorder.

Our patient commenced ERT with notable improvement in cytopenia, seizure control, and hepatosplenomegaly, though she remained dependent on hemodialysis. While ERT is highly effective in ameliorating hematological and visceral manifestations of GD, its ability to reverse established kidney impairment remains uncertain [5]. It may help slow or prevent further decline in kidney function by reducing glucocerebroside accumulation, but irreversible structural damage is unlikely to be reversed. This underscores the importance of early diagnosis and timely initiation of therapy to preserve kidney function and optimize outcomes.

### What is new?

- A unique presentation of GD in infancy with a novel genetic variant (c.1222A > T, p.Thr408Ser), initially manifesting as SRNS and progressive chronic kidney disease.

## Data Availability

The datasets generated and/or analyzed during the current study are available from the corresponding author upon reasonable request.

## References

[1] Nagral A (2014) Gaucher disease. J Clin Exp Hepatol 4:37–50 25755533 10.1016/j.jceh.2014.02.005 PMC4017182

[2] Mignot C, Doummar D, Maire I, De Villemeur TB, French Type 2 Gaucher Disease Study Group (2006) Type 2 Gaucher disease: 15 new cases and review of the literature. Brain Dev 28:39–48 16485335 10.1016/j.braindev.2005.04.005

[3] Al-Bderat J, Abbadi N, Shammari A, Al-Bderat A (2016) Gaucher disease in a patient with focal segmental glomerulosclerosis. Saudi J Kidney Dis Transpl 27:1287–1289 27900985 10.4103/1319-2442.194696

[4] Liang M, Zhu S, Liu S, Chen J, Li D, Luo C, Wang X, Jiang Z (2023) Gaucher disease in a patient with membranoproliferative glomerulonephritis: case report. BMC Nephrol 24:287 37773105 10.1186/s12882-023-03163-9 PMC10541703

[5] Hassanin F, Abbas AH, Schalaan M, Rabea M (2022) Gaucher disease: recent advances in the diagnosis and management. Med J Viral Hep 6:6–10

